# Effects of Adding Polysaccharides and Citric Acid into Sodium Dihydrogen Phosphate Mixing Solution on the Material Properties of Gelatin-Hybridized Calcium-Phosphate Cement

**DOI:** 10.3390/ma10080941

**Published:** 2017-08-12

**Authors:** Keishi Kiminami, Toshiisa Konishi, Minori Mizumoto, Kohei Nagata, Michiyo Honda, Hidetoshi Arimura, Mamoru Aizawa

**Affiliations:** 1 Department of Applied Chemistry, School of Science and Technology, Meiji University, 1-1-1 Higashimita, Tama-ku, Kawasaki 214-8571, Japan; keishi.kiminami@gunze.co.jp (K.K.); nagata_k@meiji.ac.jp (K.N.); michiyoh@meiji.ac.jp (M.H.); 2 GUNZE LIMITED, 1 Zeze, Aono-cho, Ayabe 623-8511, Japan; hidetoshi.arimura@gunze.co.jp; 3 Department of Medical Bioengineering, Graduate School of Natural Science and Technology, Okayama University, 3-1-1 Tsushima-naka, Kita-ku, Okayama 700-8530, Japan; toshiisak@cc.okayama-u.ac.jp; 4 Kanagawa Academy of Science and Technology, 3-2-1 Sakado, Takatsu-ku, Kawasaki 213-0012, Japan; mizumotomi@gmail.com

**Keywords:** gelatin particles, bioresorbability, α-tricalcium phosphate, calcium-phosphate cement, Inositol phosphate, anti-washout capability, mechanical property

## Abstract

We have succeeded in improving the material properties of a chelate-setting calcium-phosphate cement (CPC), which is composed of hydroxyapatite (HAp) the surface of which has been modified with inositol hexaphosphate (IP6) by adding α-tricalcium phosphate (α-TCP) powder. In order to create a novel chelate-setting CPC with sufficient bioresorbability, gelatin particles were added into the IP6-HAp/α-TCP cement system to modify the material properties. The effects of adding polysaccharides (chitosan, chondroitin sulfate, and sodium alginate) into the sodium dihydrogen phosphate mixing solution on the material properties of the gelatin-hybridized CPC were evaluated. The results of mechanical testing revealed that chondroitin sulfate would be the most suitable for fabricating the hybridized CPC with higher compressive strength. Moreover, further addition of an appropriate amount of citric acid could improve the anti-washout capability of the cement paste. In summary, a gelatin-hybridized IP6-HAp/α-TCP cement system prepared with a mixing solution containing chondroitin sulfate and citric acid is expected to be a beneficial CPC, with sufficient bioresorbability and material properties.

## 1. Introduction

Autografts remain the gold standard for bone reconstruction and augmentation despite some disadvantages, such as morbidity and limited availability. Among synthetic bone grafts, calcium-phosphate cements (CPCs) is promising alternative to autografts because of its biocompatibility, osteoconductivity, and injectability. In general, set CPCs are composed of hydroxyapatite (HAp). However, CPCs have some drawbacks, such as insufficient mechanical properties, low bioresorability, and weak resistance to disintegration upon early contact with blood or other physiological fluids [1,2].

One of the present authors and his coworkers have developed a novel CPC, which set on the basis of chelate-bonding of inositol hexaphosphate (IP6) to calcium ions [3,4]. This novel CPC could be fabricated by mixing HAp powder surface-modified with IP6 and suitable aqueous solution. 

Accordingly, we have recently succeeded in improving the compressive strength (CS) and anti-washout capability of the chelate-setting IP6-HAp cement by the addition of α-tricalcium phosphate (α-TCP) powder [5]. This improvement was attributed to interlocking between precipitated calcium-deficient hydroxyapatite (CDHA) crystals formed through hydrolysis of the α-TCP powder.

However, as with most conventional CPCs, the newly created IP6-HAp/α-TCP cement system lacked macroporosity to allow the infiltration of cells that could promote material resorption and new bone formation. Therefore, an effective method to create macropores inside the system should be established to enhance its bioresorbability.

One of the promising approaches to providing CPCs with macroporosity is the incorporation of biodegradable solid-state polymers, resulting in the formation of macropores along with their degradation over time [6]. In fact, the development of CPCs combined with poly (lactic acid-*co*-glycolic acid) (PLGA) [7,8,9], chitosan [10,11], or gelatin [12,13,14] have been reported by many researchers.

Of these polymeric materials, gelatin particles seemed to be the most suitable for incorporation into the IP6-HAp/α-TCP cement toward enhancement of its bioresorbability. Gelatin does not generate high amounts of acidic products during degradation, unlike PLGA, and has a higher in vivo resorption rate than PLGA [12]. In addition, some studies have shown that gelatin incorporated into CPCs could promote initial cell adhesion and proliferation, followed by activating their metabolism and differentiation [15,16]. Furthermore, some preliminary studies using canine and rabbit bone-defect models have demonstrated that 10 or 15 mass% of gelatin particles loaded into a conventional CPC could accelerate in vivo resorption of the CPC and improve bone regeneration [17,18,19]. The gelatin particles used in these studies had random size distributions with a median particle size of 200 μm that arose naturally from the manufacturing process. The creation of sufficiently interconnected macropores inside the CPC associated with the gelatin degradation was assumed to facilitate the infiltration of cells, material resorption, and subsequent new bone formation. On the basis of the results of these studies, we expected that these gelatin particles could also act as interconnected macropore-forming agents inside IP6-HAp/α-TCP cement, towards enhancement of its bioresorbability. 

However, the incorporation of gelatin reportedly tended to negatively affect the material properties of CPCs, in respect of initial setting time (IST) and mechanical strength, due to interference of the hardening reaction [20,21]. Therefore, these material properties of IP6-HAp/α-TCP cement required some kind of compensating strategies. 

To address this issue, the addition of polysaccharides (especially, chitosan [2], sodium alginate [22], and chondroitin sulfate [23]) into the mixing solution (sodium dihydrogen phosphate solution) for cement preparation would be beneficial. This is because the material properties as well as the handling properties and injectability of the CPC could be expected to improve based upon the polysaccharides’ ability to form a viscous gel [6]. In particular, it was hypothesized that the IST and anti-washout capability of IP6-HAp/α-TCP cement paste would not be significantly influenced by the incorporation of gelatin particles because of enhanced handling properties. Moreover, we expected that the polysaccharides could contribute to maintaining the CS of the cement specimens during gelatin incorporation, on the basis of interactions between the polysaccharides, calcium ions, and gelatin particles.

As an additional strategy, citric acid was employed as a supplementary additive to the mixing solution containing polysaccharides. Citric acid reportedly promotes the hardening reaction and increases the mechanical strength of CPCs by means of a chelating reaction with calcium ions [24,25]. Therefore, further modification of the material properties of the gelatin-hybridized CPC was expected to be achieved by the addition of citric acid.

In view of the optimization of the mixing solution for the gelatin-hybridized IP6-HAp/α-TCP cement, the aim of the present study was to evaluate the material properties of the cement pastes and set specimens and the effect of adding polysaccharides (chitosan, chondroitin sulfate, and sodium alginate) and citric acid to the mixing solution (sodium dihydrogen phosphate solution).

Finally, we briefly describe the highlights of this work. The results of our evaluations demonstrated that chondroitin sulfate was more effective than the other polysaccharides, from the viewpoint of preparing the CPC with higher CS. Moreover, the addition of an appropriate amount of citric acid into the mixing solution containing chondroitin sulfate was found to further enhance the CS and the anti-washout capability of the CPC.

## 2. Materials and Methods

### 2.1. Preparation of α-TCP Powder

Ten grams of commercially available α-TCP raw powder (*α-TCP*; Taihei Chemical Industrial Co., Ltd., Osaka, Japan) were used as the starting material. The α-TCP raw powder was ground for 60 min at a rotation rate of 300 rpm, using a planetary ball mill (Pulverisette 6, Fritsch, Idar-oberstein, Germany) in a ZrO_2_ pot with 180 g of ZrO_2_ beads 2 mm in diameter, under wet conditions (40 cm^3^ of distilled water). After ball-milling, the resulting slurry was filtered and freeze-dried for 24 h to yield the ball-milled α-TCP powders.

### 2.2. Preparation of HAp Powder Surface-Modified with IP6

IP6-Na solution at a concentration of 8000 ppm was prepared using 50 mass% phytic acid (Wako Pure Chemical Industries, Ltd., Osaka, Japan) and adjusted to a pH of 7.3 with NaOH solution. Ten grams of commercially-available HAp powder (HAp-100; Taihei Chemical Industrial Co., Ltd., Osaka, Japan) were simultaneously ground and surface-modified by ball-milling with 50 cm^3^ of the 8000 ppm IP6-Na solution, using the planetary ball mill for 60 min at a rotation rate of 300 rpm in a ZrO_2_ pot with 180 g of ZrO_2_ beads 2 mm in diameter. After ball-milling, the resulting slurry was filtered and freeze-dried for 24 h to yield the IP6-HAp powder.

### 2.3. Preparation and Characterization of Gelatin Particles

Gelatin particles extracted from porcine skin were alkaline-processed, ground (Nitta Gelatin Inc., Osaka, Japan) and heated at 140 °C for 14 h under vacuum conditions. The thermally cross-linked gelatin particles were irradiated by γ-ray (25 kGy). The morphology of the prepared gelatin particles was observed by scanning electron microscopy (SEM; TM-1000, Hitachi High-Technologies Corporation, Tokyo, Japan) at an accelerating voltage of 15 kV. The particle size distribution of the gelatin particles was determined by a laser particle size analyzer (LMS-3000, Seishin Enterprise Co., Ltd., Tokyo, Japan).

### 2.4. Preparation of Mixing Solutions for Cement Fabrication

As shown in Table 1, the mixing solutions containing polysaccharides (chitosan, chondroitin 6-sulfate sodium salt, and sodium alginate), sodium dihydrogen phosphate, and citric acid were prepared and adjusted to a pH of 7.0 with NaOH solution. With the exception of chitosan (Daichitosan Coat GL, Dainichiseika Color & Chemicals Mfg. Co., Ltd., Tokyo, Japan), the chemicals were purchased from Wako Pure Chemical Industries, Ltd., Osaka, Japan.

### 2.5. Preparation of the Cement Pastes Hybridized with the Gelatin Particles

The IP6-HAp powder was premixed with the prepared α-TCP powder at a mixing ratio of 20/80 (g/g) for 5 min using Super Micro V-Shape Mixer (MC, Tsutsui Scientific Instruments Co., Ltd., Japan). The premixed powder was further mixed with the gelatin particles for an additional 5 min at a ratio of 100/0, 95/5, 90/10, or 85/15 (g/g). The cement pastes, hybridized with various amounts of the gelatin particles, were prepared by mixing the obtained powder and the mixing solutions listed in Table 1 at the designated powder/liquid (P/L) ratios (g/cm^3^) shown in Table 2.

### 2.6. Evaluation of the Cement Pastes and Set Specimens

The initial setting time (IST) was measured using a light Gillmore needle (113.4 g) in accordance with JIS T 0330-4. The prepared cement pastes were packed in plastic molds (8 mm in diameter and 2 mm in height), and the IST was measured at the desired periods until the cement pastes were set. Three cement specimens were tested to obtain an average value with standard deviation.

For the compressive strength (CS) test, the set cement pastes were packed in cylindrical Teflon^®^ molds (6 mm in diameter and 12 mm in height), maintained at 37 °C at 100% humidity for 1 h, and kept in distilled water at 37 °C for 24 h. The CS tests were performed on the cement specimens using a universal testing machine (AG-5KNXplus, Shimadzu Co., Kyoto, Japan) in accordance with JIS T 0330-4. The crosshead speed was 0.5 mm/min, and a load cell of 5 kN was used. Four cement specimens were tested to obtain an average value with standard deviation. The fracture surfaces of the cement specimens were observed by the SEM. The samples were sputter-coated with Au prior to the observation.

For the observation of macropores formed through removal of the gelatin particles, the prepared cement paste, hybridized with 10 mass% of gelatin particles and fabricated with Chondro-1.0, was packed in the cylindrical Teflon^®^ mold, maintained at 37 °C at 100% humidity for 24 h, and placed in a muffle furnace (KDF S-100, Denken-Highdental Co., Ltd., Kyoto, Japan) at 500 °C for 5 h to burn out the gelatin particles inside the cement specimen. The fractured surface of the specimen was observed by the SEM after the sputter-coating with Au.

The X-ray diffraction (XRD) patterns of the cement specimens after the CS tests were measured with an X-ray diffractometer (XRD-6100, Shimadzu Co., Kyoto, Japan) equipped with a CuKα radiation source. The crystal phase was identified with respect to the JCPDS reference patterns for HAp (#09-0432). 

The anti-washout capability tests were also conducted in accordance with JIS T 0330-4. At 5 min after mixing the cement pastes, the cement pastes (0.3 cm^3^) were pushed out by syringes onto wire gauze. Subsequently, the cement pastes were immersed into saline (30 cm^3^) with the wire gauze. After 24 h immersion at 37 °C, the cement specimens on the wire gauze and the washed-out specimens that had seeped through the gauze were taken out and dried at 60 °C for 24 h, respectively. Washout ratios were calculated according to the following equation:

(1) $$\mathrm{Washout}\;\mathrm{ratio}\;\left(\%\right)=\frac{W_{B}}{W_{A}\;+\;W_{B}}\times 100$$

where *W*_A_ represents the weight of the cement specimens on the wire gauze and *W*_B_ represents the weight of the washed-out cement specimens under the wire gauze. Four cement specimens were tested to obtain an average value with standard deviation.

## 3. Results and Discussion

### 3.1. Characterization of the Prepared Gelatin Particles

**Figure 1:**
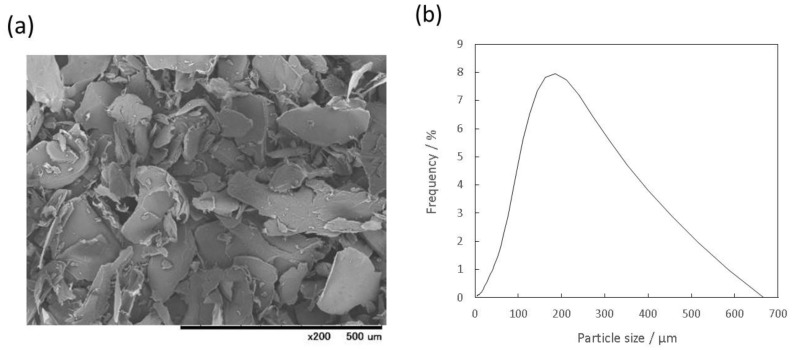
(**a**) Scanning electron microscopy (SEM) micrograph and (**b**) particle-size distribution of the prepared gelatin particles as macropore-forming agents.

A SEM micrograph of the prepared gelatin particles show that these particles had irregular shapes (Figure 1a). Figure 1b shows that the particle size distribution of the gelatin particles was broad, in the range of 5 to 666 μm, and the median particle size was 184 μm. As mentioned above, the wide range of size distribution and irregular shapes of the gelatin particles were expected to be beneficial for the creation of interconnected macropores and subsequent cell migration. With respect to the median particle size, Gauthier et al. reported that macropores larger than 100 μm could promote cell colonization and bone ingrowth [26]. Thus, the median particle size was considered to be suitable for us to achieve enhancement of bioresorption of the IP6-HAp/α-TCP cement and subsequent new bone formation.

### 3.2. Effects of Additions of Polysaccharides into the Mixing Solution on Material Properties of the Gelatin-Hybridized Cement

**Figure 2:**
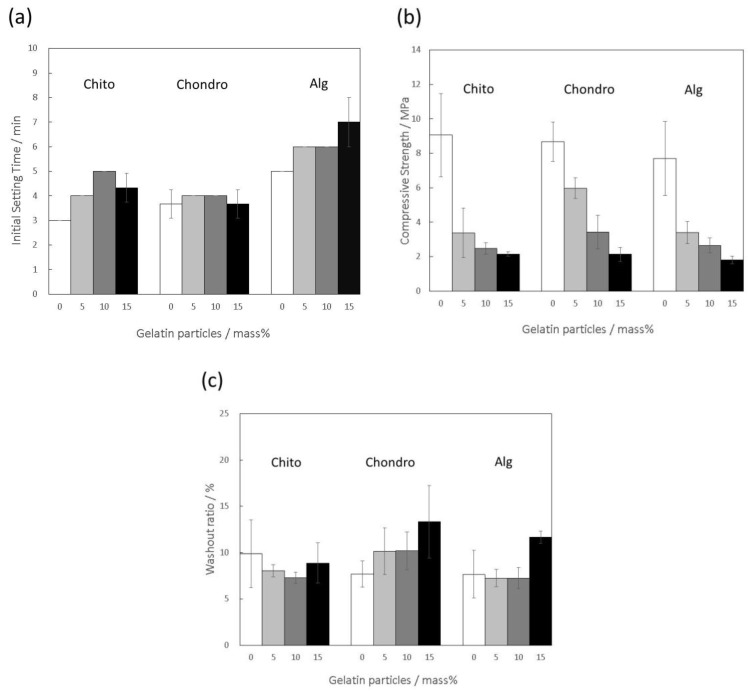
(**a**) Initial setting time; (**b**) Compressive strength; and (**c**) Washout ratio of the 0–15 mass% of gelatin-hybridized hydroxyapatite powder surface-modified with inositol hexaphosphate mixed with α-tricalcium phosphate powder (IP6-HAp/α-TCP) cement specimens prepared with mixing solutions, Chito, Chondro, and Alg, listed in Table 1, at the mixing ratios shown in Table 2.

The effects of adding polysaccharides into the mixing solution (sodium dihydrogen phosphate solution) on the material properties of the gelatin-hybridized IP6-HAp/α-TCP cement was evaluated using the mixing solutions Chito, Alg, and Chondro, as listed in Table 1. Figure 2a shows that the IST of the cement pastes prepared with Chito or Alg increased with the amount of gelatin particles. These results are in agreement with a previous report indicating that incorporation of gelatin into a CPC prolonged the setting time due to the adverse effect of gelatin on the hardening reaction of the CPC [21]. Interestingly, as we expected, the IST of the pastes was little affected by the gelatin hybridization when Chondro was used for the cement preparation. In addition to the constant P/L ratios among the pastes, this result might be caused by electrostatic interaction between the negatively charged sulfate group of chondroitin sulfate and calcium ions or the gelatin particles [27]. Nevertheless, all of the cement pastes tested here satisfied the following requirement for favorable handling in clinical applications [28]:

3 min ≤ IST < 8 min(2)

Figure 2b demonstrates that, against our expectation as mentioned above, the CS of the hybridized cement specimens fell significantly with increasing amounts of gelatin particles, due to the weaker gelatin phase, no matter which polysaccharide was used in the mixing solutions. Actually, the CS declined to approximately 2 MPa with the incorporation of 15 mass% of the gelatin particles, regardless of the polysaccharides. Mosekilde et al. showed that the CS of a human whole vertebral body was in the range of 1.5–7.8 MPa [29], indicating that artificial bone grafts for vertebroplasty require an initial CS around this range. Therefore, when considering clinical applications in vertebroplasty, it seems to be necessary to have the incorporation ratio of the gelatin particles lower than 15 mass%, though bioresorbability of the CPC might be decreased. Meanwhile, when 5 or 10 mass% of gelatin particles were hybridized, Chondro provided the CPC with comparatively higher CS values of 6.0 MPa and 3.4 MPa, respectively. Along with the relatively higher P/L ratios, the interaction between chondroitin sulfate and calcium ions or gelatin particles, as mentioned above, was likely the main contributor to the enhancement of the CS.

Figure 2c indicates that the washout ratios of the cement paste prepared with Chondro or Alg tended to increase with the amount of the gelatin particles, despite our expectation that the anti-washout properties would be maintained during gelatin hybridization. These results were presumably due to swelling of the gelatin particles, which allowed water penetration into the cement pastes. However, when Chito was used for the cement preparation, the washout ratio of the cement paste was not significantly influenced by the gelatin hybridization, as we expected. In addition to the formation of viscous sol preventing water penetration, as shown in a previous report [2], the generation of the polyelectrolyte complex between the cation-charged chitosan and the gelatin particles might contribute to resisting washout [30].

**Figure 3:**
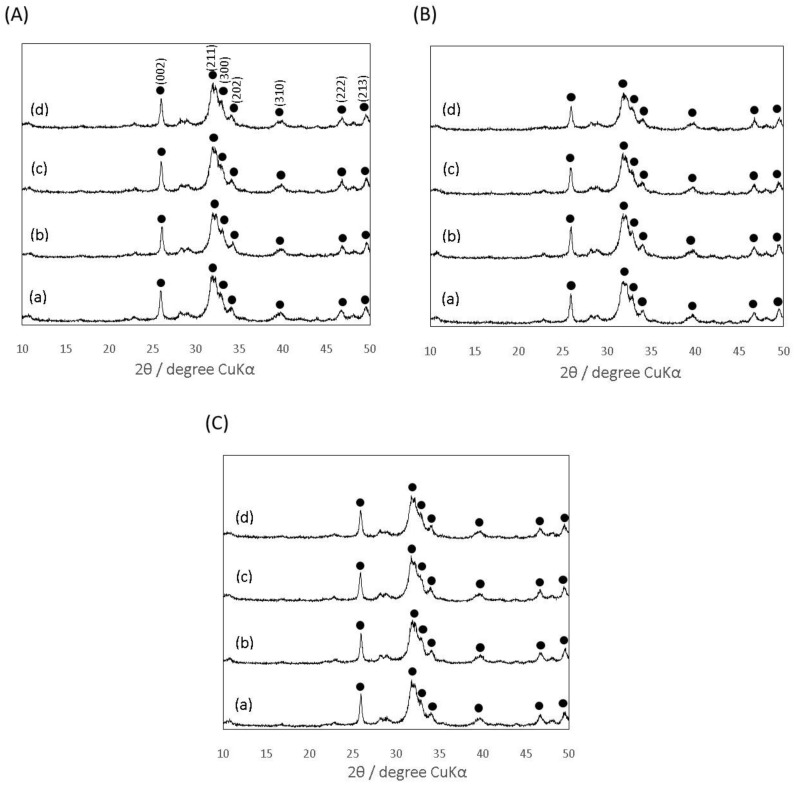
X-ray diffraction (XRD) patterns of the IP6-HAp/α-TCP cement specimens prepared with the three kinds of mixing solutions: (**A**) Chito; (**B**) Chondro; and (**C**) Alg, hybridized with (a) 0, (b) 5, (c) 10, and (d) 15 mass% of the gelatin particles, respectively. Closed circles indicated typical HAp peaks.

The XRD patterns of the cement specimens after the CS test reveals that the gelatin hybridization little affected their crystalline phase, regardless of the polysaccharides used (Figure 3). All of the set cement specimens exhibited broad XRD patterns and obvious diffraction peaks around 2θ = 32°, derived from the low crystalline apatite phase. Ginebra et al. reported that extent of conversion of α-TCP with a specific surface area of 1.0 m^2^/g to CDHA was nearly 80% after immersion in Ringer’s solution at 37 °C for 24 h [31]. The difference in the extent of conversion was probably due to the larger specific surface area (6.3 m^2^/g) of the α-TCP powder. In addition, calcium chloride contained in Ringer’s solution might retard the conversion providing the common ions related with the hydrolysis reaction, as Monma et al. previously reported [32].

As mentioned above, chondroitin sulfate would be the most suitable addition into the mixing solution among the three polysaccharides in order to fabricate a 5 or 10 mass% of gelatin-hybridized CPC with higher CS.

**Figure 4:**
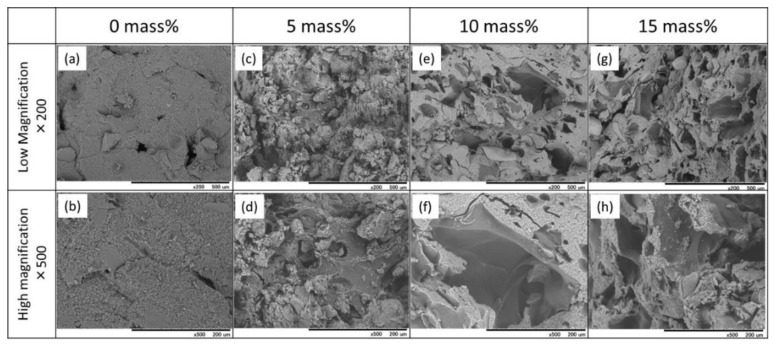
SEM micrographs of the fractured surfaces of the IP6-HAp/α-TCP cement specimens hybridized with (**a**,**b**) 0; (**c**,**d**) 5; (**e**,**f**) 10; and (**g**,**h**) 15 mass% of the gelatin particles prepared with mixing solution Chondro.

SEM micrographs of the fractured surfaces of the cement specimens, with and without gelatin hybridization, prepared with Chondro are shown in Figure 4. The cement specimen without gelatin particles exhibited a smooth appearance with entangled particles. On the other hand, the gelatin-hybridized cement specimens showed a relatively rough appearance, due to the presence of the gelatin particles.

### 3.3. Effects of Addition of Citric Acid into the Mixing Solution Containing Chondroitin Sulfate on Material Properties of the Gelatin-Hybridized Cement

In order to further modify the material properties of the gelatin-hybridized cement, we evaluated the effects of adding various concentrations of citric acid into the mixing solution Chondro on the material properties of the CPCs.

**Figure 5:**
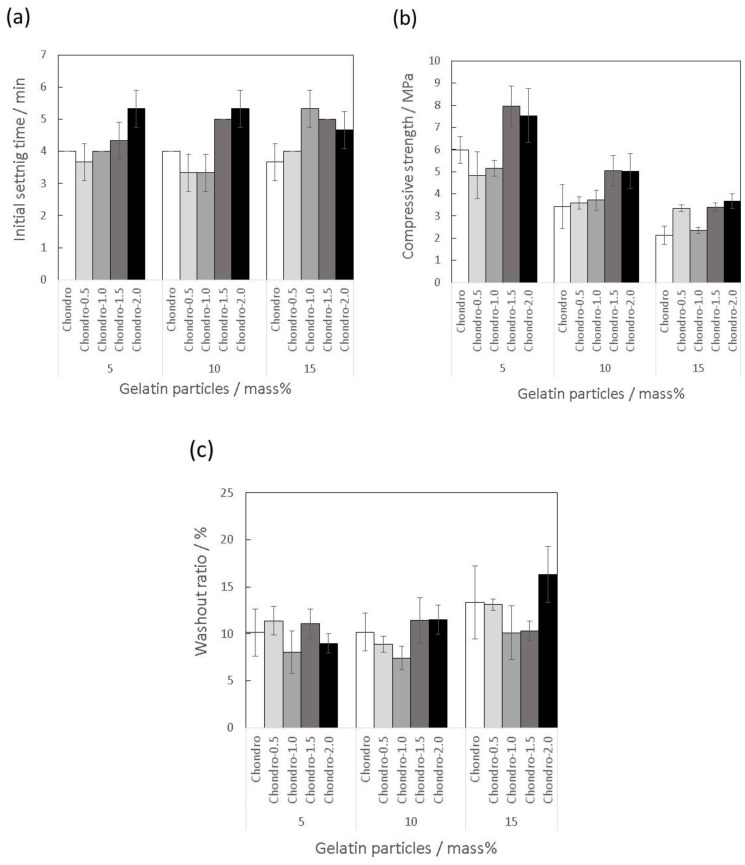
(**a**) Initial setting time, (**b**) Compressive strength, and (**c**) Washout ratio of the gelatin-hybridized IP6-HAp/α-TCP cement specimens prepared with the various mixing solutions—Chondro, Chondro-0.5, Chondro-1.0, Chondro-1.5, and Chondro-2.0—listed in Table 1, at the mixing ratios shown in Table 2.

Figure 5a shows that increasing the concentration of citric acid tended to prolong initial setting of the cement pastes, regardless of the amount of the gelatin particles, while still satisfying the requirement for IST mentioned above. These results are consistent with a previous report indicating that the chelate effect between citric acid and calcium ions could inhibit the processes of nucleation and growth of CDHA crystals onto the α-TCP particles [25].

Figure 5b demonstrates that the addition of citric acid led to enhancement of the CS, regardless of the degree of gelatin hybridization, as we hypothesized. In particular, using Chondro-1.5 or Chondro-2.0 enhanced the CS of the 15 mass% of gelatin-hybridized CPC to the same level as that of the 10 mass% of gelatin-hybridized CPC prepared with Chondro. Furthermore, these two mixing solutions could also increase the CS of the 5 or 10 mass% of gelatin-hybridized CPC to 7.5–8.0 MPa or 5.0 MPa, which is in the range of that of human vertebral body [29]. These results are likely attributable to increased P/L ratios and reduced air bubbles inside the cement specimens, associated with improved injectability of the cement pastes. It has been suggested that citric acid molecules could wrap themselves around the α-TCP particles and give them a highly-negative charge, which results in enhancing the injectability of the cement pastes [25].

Figure 5c reveals that the cement paste hybridized with 15 mass% of the gelatin particles prepared with Chondro-2.0 had a relatively high washout ratio. This result might be due to interference with the formation of CDHA crystals induced by citric acid, allowing water penetration into the cement paste. Meanwhile, the cement paste hybridized with 15 mass% of gelatin particles obtained comparatively low washout ratios when Chondro-1.0 or Chondro-1.5 was used. Considering the difference in the CS, Chondro-1.5 is thought to be the most suitable for fabricating the 15 mass% of gelatin-hybridized CPC. On the other hand, the CPC hybridized with 10 mass% of gelatin particles obtained the lowest washout ratio when Chondro-1.0 was used. It could be concluded that Chondro-1.0 would be the most favorable for fabricating a 10 mass% gelatin-hybridized CPC when the balance between the CS and the washout ratio is taken into account. With respect to 5 mass% gelatin-hybridized CPC, Chondro-2.0 is likely to be optimum in terms of the highest CS and relatively low washout ratio.

Among the three optimized CPCs differing in the amount of hybridized gelatin particles and the composition of the mixing solutions, the 10 mass% gelatin-hybridized IP6-HAp/α-TCP cement prepared with Chondro-1.0 is the most promising CPC due to its reduced washout ratio, modest CS, and expected bioresorbability.

**Figure 6:**
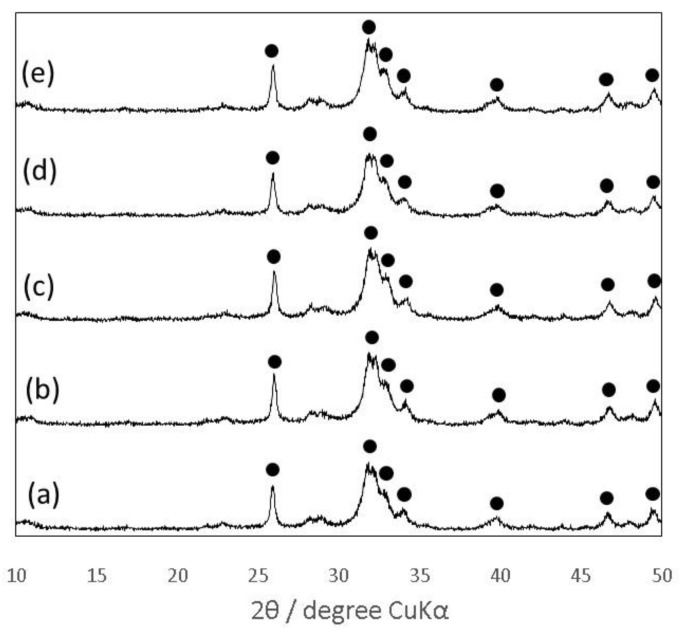
XRD patterns of the IP6-HAp/α-TCP cement specimens hybridized with 10 mass% of the gelatin particles prepared with the mixing solutions: (**a**) Chondro; (**b**) Chondro-0.5; (**c**) Chondro-1.0; (**d**) Chondro-1.5; and (**e**) Chondro-2.0. Closed circles indicated typical HAp peaks.

**Figure 7:**
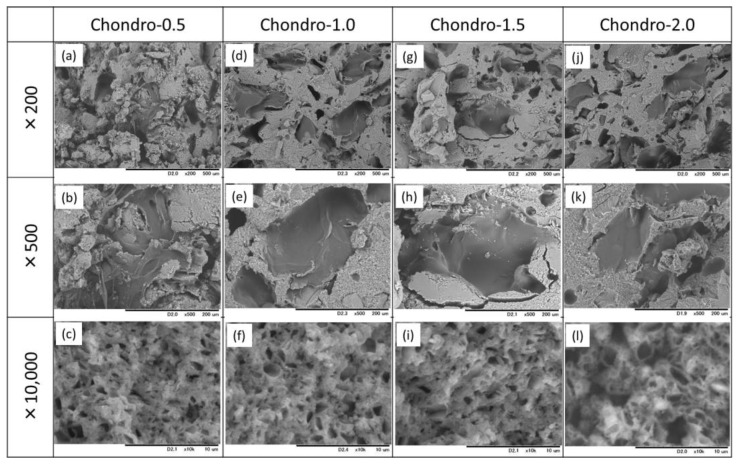
SEM micrographs of the fractured surfaces of the IP6-HAp/α-TCP cement specimens hybridized with 10 mass% of the gelatin particles prepared with the mixing solutions: (**a**–**c**) Chondro-0.5; (**d**–**f**) Chondro-1.0; (**g**–**i**) Chondro-1.5; and (**j**–**l**) Chondro-2.0.

Figure 6 shows that the addition of citric acid into the mixing solution Chondro did not significantly affect the XRD patterns of the 10 mass% gelatin-hybridized cement specimens. Furthermore, the fractured surfaces of all cement specimens exhibited similar morphologies, and entanglement of needle-shaped apatite crystals were observed (Figure 7).

**Figure 8:**
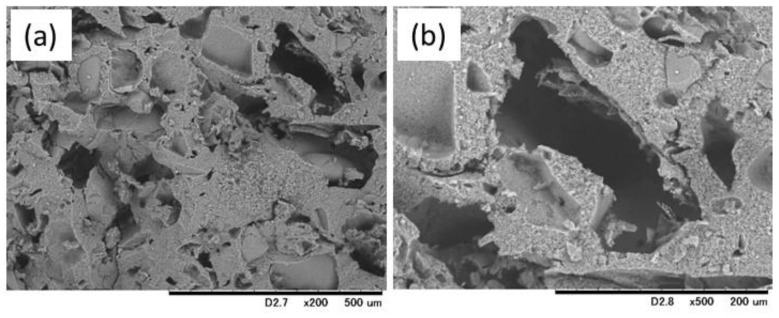
SEM micrographs at (**a**) lower and (**b**) higher magnification of the fractured surface of the 10 mass% gelatin-hybridized IP6-HAp/α-TCP cement specimen prepared with mixing solution Chondro-1.0, in which the gelatin particles were burned out to create macropores.

Figure 8 demonstrates that irregularly shaped macropores were successfully formed inside the cement specimen after removal of the gelatin particles. This result indicates that the equivalent macropores are expected to be created by degradation of the gelatin particles when the specimen is implanted into bone defects.

### 3.4. Summary

In summary, chondroitin sulfate was found to be a more beneficial additive to the mixing solution than chitosan and sodium alginate for fabricating the 5 or 10 mass% of gelatin-hybridized IP6-HAp/α-TCP cement with higher CS. This is probably attributable to interaction between the negatively charged sulfonyl group of chondroitin sulfate and calcium ions or gelatin particles. Furthermore, it was demonstrated that the addition of an appropriate amount of citric acid into the chondroitin sulfate mixing solution could enhance the CS and the anti-washout capability of the hybridized CPCs. These results are presumably due to improved injectability induced by the high negative charges of α-TCP particles wrapped with citric acid. In particular, the 10 mass% gelatin-hybridized cement prepared with the mixing solution containing chondroitin sulfate and 1.0 mass% citric acid was considered the most promising CPC because of its comparatively good balance between CS, anti-wash-out capability, and expected bioresorbability.

## 4. Discussion

CPCs are common as artificial bone grafts applicable to minimally-invasive surgery due to their injectabilily, biocompatibility, and osteoconductivity. Meanwhile, they have to satisfy a wide variety of required specifications to be used in various clinical fields (e.g., orthopedics and dentistry) as an alternative to autografts. First of all, CPCs are required not to cause inflammation in the host tissues. In addition, they need to be equipped with specific material properties, such as initial setting time, anti-washout capability, appropriate mechanical strength, along with bioresorbability and bone-forming ability in a proper balance. However, the CPC with ideal material properties, including bioresorbability and bone-forming ability, has not yet been developed.

Within this context, we previously developed the chelate-setting CPC, IP6-HAp/α-TCP cement. This CPC had a major advantage over most conventional CPCs in that its setting mechanism was independent of an acid-base reaction, which might cause inflammation in the surrounding tissues [33]. Furthermore, it was demonstrated that the chelate-setting CPC set within a few minutes, and had the anti-washout capability and the CS equivalent to cancellous bones. Accordingly, we expected enhancement of bioresorbability of this CPC as one of effective strategies to develop the ideal CPC.

In the present study, with the aim of establishing fundamental material design for such the ideal CPC, we incorporated the gelatin particles into IP6-HAp/α-TCP cement to enhance its bioresorbability and bone-forming ability. At the same time, composition of the mixing solution was optimized from the viewpoint of mitigating the effects of the gelatin-hybridization on material properties of the chelate-setting CPC. 

In particular, we evaluated effects of polysaccharides contained in the mixing solution on the material properties of the gelatin-hybridized CPC. As the result, two important findings were obtained. Firstly, the washout ratio of the cement paste prepared with the mixing solution containing chitosan was not significantly affected by the gelatin-hybridization. This finding suggests that the gelatin-hybridized CPC prepared with the mixing solution containing chitosan might be beneficial in certain clinical applications (e.g., dental treatment) where anti-washout capability is required rather than CS. Meanwhile, it was revealed that use of chondroitin sulfate provided the gelatin-hybridized CPC with higher CS. This was the other important finding because CS is one of fundamental requirements to avoid risks related to corruption of the cement specimen after implantation into bone tissues.

Moreover, we demonstrated that the addition of an appropriate amount of citric acid into the mixing solution containing chondroitin sulfate could modify material properties of the gelatin-hybridized CPC. In fact, the amounts of citric acid added into the mixing solution to prepare 5, 10, and 15 mass% gelatin-hybridized CPC could be optimized to 2.0, 1.0, and 1.5 mass% respectively in consideration of the material properties. With a balance between the material properties and expected bioresorbability and bone-forming ability, it could be concluded that 10 mass% gelatin-hybridized IP6-HAp/α-TCP cement prepared with the mixing solution containing chondroitin sulfate and 1.0 mass% citric acid would be the fundamental design for the ideal CPC.

## Figures and Tables

**Table 1:** Compositions of the mixing solutions to prepare cement pastes.

Abbreviation	Chitosan (Mass%)	Sodium Alginate (Mass%)	Chondroitin 6-Sulfate Sodium Salt (Mass%)	Citric Acid (Mass%)	Sodium Dihydrogen Phosphate (Mass%)
Chito	10	0	0	0	2.5
Alg	0	1.0	0	0	2.5
Chondro	0	0	15	0	2.5
Chondro-0.5	0	0	15	0.5	2.5
Chondro-1.0	0	0	15	1.0	2.5
Chondro-1.5	0	0	15	1.5	2.5
Chondro-2.0	0	0	15	2.0	2.5

**Table 2:** P/L ratios (g/cm^3^) for cement fabrication.

Abbreviation	Amount of the Hybridized Gelatin Particles (Mass%)			
	0	5	10	15
Chito	1/0.70	1/0.82	1/0.82	1/0.82
Alg	1/0.70	1/0.70	1/0.82	1/0.94
Chondro	1/0.70	1/0.70	1/0.70	1/0.70
Chondro-0.5	-----	1/0.70	1/0.70	1/0.74
Chondro-1.0	-----	1/0.70	1/0.70	1/0.70
Chondro-1.5	-----	1/0.62	1/0.62	1/0.70
Chondro-2.0	-----	1/0.62	1/0.62	1/0.60

## References

[1] Wang X., Chen L., Xiang H., Ye J. (2007). Influence of anti-washout agents on the rheological properties and injectability of a calcium phosphate cement. J. Biomed. Mater. Res. Part B.

[2] Takeuchi M., Miyamoto Y., Ishikawa K., Toh T., Yuasa T., Nagayama M., Suzuki K. (1998). Initial histological evaluation of anti-washout type fast-setting calcium phosphate cement following subcutaneous implantation. Biomaterials.

[3] Aizawa M., Haruta Y., Okada I. (2003). Development of novel cement processing using hydroxyapatite particles modified with inositol phosphate. Arch. Bioceram. Res..

[4] Kida K., Horiguchi Y., Oribe K., Morisue H., Matsumoto M., Toyama Y., Aizawa M. (2008). Biological evaluation of chelate-setting apatite cement using inositol phosphate. Key Eng. Mater..

[5] Kiminami K., Matsuoka K., Konishi T., Mizumoto M., Honda M., Arimura H., Aizawa M. (2017). Effects of addition of α-tricalcium phosphate powders on material properties of the chelate-setting hydroxyapatite cement. Phosphorus Res. Bull..

[6] Perez R.A., Kim H.W., Ginebra M.P. (2012). Polymeric additives to enhance the functional properties of calcium phosphate cements. J. Tissue Eng..

[7] Habraken W.J.E.M., Liao H.B., Zhang Z., Wolke J.G.C., Grijpma D.W., Mikos A.G., Feijen J., Jansen J.A. (2010). In vivo degradation of calcium phosphate cement incorporated into biodegradable microspheres. Acta Biomater..

[8] Habraken W.J.E.M., Wolke J.G.C., Mikos A.G., Jansen J.A. (2006). Injectable PLGA microsphere/calcium phosphate cements: Physical properties and degradation characteristics. J. Biomater. Sci. Polym. Ed..

[9] Ruhé P.Q., Hedberg-Dirk E.L., Padron N.T., Spauwen P.H.M., Jansen J.A., Mikos A.G. (2006). Porous poly(DL-lactic-*co*-glycolic acid)/calcium phosphate cement composite for reconstruction of bone defects. J. Tissue Eng..

[10] Lian Q., Li D.C., He J.K., Wang Z. (2008). Mechanical properties and in vivo performance of calcium phosphate cement-chitosan fibre composite. Proc. Inst. Mech. Eng. Part H.

[11] Muzzarelli C., Muzzarelli R.A.A. (2002). Natural and artificial chitosan-inorganic composites. J. Inorg. Biochem..

[12] Habraken W.J.E.M., Wolke J.G.C., Mikos A.G., Jansen J.A. (2009). Porcine gelatin microsphere/calcium phosphate cement composites: An in vitro degradation study. J. Biomed. Mater. Res. Part B.

[13] Link D.P., van den Dolder J., van den Beucken J.J.J.P., Habraken W., Soede A., Boerman O.C., Mikos A.G., Jansen J.A. (2009). Evaluation of an orthotopically implanted calcium phosphate cement containing gelatin microparticles. J. Biomed. Mater. Res. Part A.

[14] Habraken W.J.E.M., de Jonge L.T., Wolke J.G.C., Yubao L., Mikos A.G., Jansen J.A. (2008). Introduction of gelatin microspheres into an injectable calcium phosphate cement. J. Biomed. Mater. Res. Part A.

[15] Bigi A., Panzavolta S., Sturba L., Torricelli P., Fini M., Giardino R. (2006). Normal and osteopenic bone-derived osteoblast response to a biomimetic gelatin-calcium phosphate bone cement. J. Biomed. Mater. Res. Part A.

[16] Perez R.A., Valle S.D., Altankov G., Ginebra M.P. (2011). Porous hydroxyapatite and gelatin/hydroxyapatite microspheres obtained by calcium phosphate cement emulsion. J. Biomed. Mater. Res. Part B.

[17] Matsumoto G., Sugita Y., Kubo K., Yoshida W., Ikada Y., Sobajima S., Neo M., Maeda H., Kinoshita Y. (2013). Gelatin powders accelerate the resorption of calcium phosphate cement and improve healing in the alveolar ridge. J. Biomater. Appl..

[18] Yomoda M., Sobajima S., Kasuya A., Neo M. (2015). Calcium phosphate cement—Gelatin powder composite testing in canine models: Clinical implications for treatment of bone defects. J. Biomater. Appl..

[19] Kasuya A., Sobajima S., Kinoshita M. (2012). In vivo degradation and new bone formation of calcium phosphate cement-gelatin powder composite related to macroporosity after in situ gelatin degradation. J. Orthop. Res..

[20] Chiang T.Y., Ho C.C., Chen D.C.H., Lai M.H., Ding S.J. (2010). Physicochemical properties and biocompatibility of chitosan oligosaccharide/gelatin/calcium phosphate hybrid cements. Mater. Chem. Phys..

[21] Fujishiro Y., Takahashi K., Sato T. (2001). Preparation and compressive strength of α-tricalcium phosphate/gelatin gel composite cement. J. Biomed. Mater. Res. Part A.

[22] Ishikawa K., Miyamoto Y., Takeuchi M., Toh T., Kon M., Nagayama M., Asaoka K. (1997). Non-decay type fast-setting calcium phosphate cement: Hydroxyapatite putty containing an increased amount of sodium alginate. J. Biomed. Mater. Res. Part A.

[23] Tamimi-Mariño F., Mastio J., Rueda C., Blanco L., López-Cabarcos E. (2007). Increase of the final setting time of brushite cements by using chondroitin 4-sulfate and silica gel. J. Mater. Sci. Mater. Med..

[24] Sarda S., Fernández E., Nilsson M., Balcells M., Planell J.A. (2002). Kinetic study of citric acid influence on calcium phosphate bone cements as water-reducing agent. J. Biomed. Mater. Res. Part A.

[25] Yokoyama A., Yamamoto S., Kawasaki T., Kohgo T., Nakatsu M. (2002). Development of calcium phosphate cement using chitosan and citric acid for bone substitute materials. Biomaterials.

[26] Gauthier O., Bouler J.M., Aguado E., Pilet P., Daculsi G. (1998). Macroporous biphasic calcium phosphate ceramics: Influence of macropore diameter and macroporosity percentage on bone ingrowth. Biomaterials.

[27] Schneiders W., Reinstorf A., Biewener A., Serra A., Grass R., Kinscher M., Heineck J., Rehberg S., Zwipp H., Rammelt S. (2009). In vivo effects of modification of hydroxyapatite/collagen composites with and without chondroitin sulphate on bone remodeling in the sheep tibia. J. Orthop. Res..

[28] Dorozhkin S.V. (2011). Self-setting calcium orthophosphate formulations: Cements, concretes, pastes and putties. Int. J. Mater. Chem..

[29] Mosekilde L., Mosekilde L. (1986). Normal vertebral body size and compressive strength: Relations to age and to vertebral and iliac trabecular bone compressive strength. Bone.

[30] Huang Y., Onyeri S., Siewe M., Moshfegheian A., Madihally S.V. (2005). In vitro characterization of chitosan-gelatin scaffolds for tissue engineering. Biomaterials.

[31] Ginebra M.P., Fernández E., de Maeyer E.A.P., Verbeeck R.M.H., Boltong M.G., Ginebra J., Driessens F.C.M., Planell J.A. (1997). Setting reactions and hardening of an apatitic calcium phosphate cement. J. Dent. Res..

[32] Monma H., Goto M., Kohmura T. (1984). Effect of additives on hydration and hardening of tricalcium phosphate. Gypsum Lime.

[33] Miyamoto Y., Ishikawa K., Takeuchi M., Toh T., Yuasa T., Nagayama M., Suzuki K. (1999). Histological and compositional evaluations of three types of calcium phosphate cements when implanted in subcutaneous tissue immediately after mixing. J. Biomed. Mater. Res. Part A.

